# Dual roles of c-Myc in the regulation of *hTERT* gene

**DOI:** 10.1093/nar/gku721

**Published:** 2014-08-28

**Authors:** Yuanjun Zhao, De Cheng, Shuwen Wang, Jiyue Zhu

**Affiliations:** 1 Department of C & M Physiology, Pennsylvania State University College of Medicine, Hershey, Pennsylvania, USA; 2 Department of Pharmaceutical Sciences, Washington State University College of Pharmacy, Spokane, Washington, USA

## Abstract

Human telomerase gene *hTERT* is important for cancer and aging. hTERT promoter is regulated by multiple transcription factors (TFs) and its activity is dependent on the chromatin environment. However, it remains unsolved how the interplay between TFs and chromatin environment controls hTERT transcription. In this study, we employed the recombinase-mediated BAC targeting and BAC recombineering techniques to dissect the functions of two proximal E-box sites at −165 and +44 nt in regulating the hTERT promoter in the native genomic contexts. Our data showed that mutations of these sites abolished promoter binding by c-Myc/Max, USF1 and USF2, decreased hTERT promoter activity, and prevented its activation by overexpressed c-Myc. Upon inhibition of histone deacetylases, mutant and wildtype promoters were induced to the same level, indicating that the E-boxes functioned to de-repress the hTERT promoter and allowed its transcription in a repressive chromatin environment. Unexpectedly, knockdown of endogenous c-Myc/Max proteins activated hTERT promoter. This activation did not require the proximal E-boxes but was accompanied by increased promoter accessibility, as indicated by augmented active histone marks and binding of multiple TFs at the promoter. Our studies demonstrated that c-Myc/Max functioned in maintaining chromatin-dependent repression of the *hTERT* gene in addition to activating its promoter.

## INTRODUCTION

The proliferative lifespan of most human somatic cells is restricted by telomeres, which serve as protective caps of chromosomal ends. Telomeric TTAGGG repeats are replenished by telomerase (1), a ribonucleoprotein complex consisting of a catalytic reverse transcriptase (TERT), an RNA template (TERC) and accessory proteins (2,3). In most human tissues, with the exception of certain stem cells, telomerase activity is either absent or very low (4,5). As a result, somatic cells suffer telomere attrition upon successive divisions and are destined to senescence. Whereas TERC is abundant in most human tissues (6), the *hTERT* gene is tightly regulated and its expression correlated with telomerase activity (7). Ectopic hTERT expression in many cell types results in telomere stabilization and cellular immortalization (8,9).

**Figure 1.**
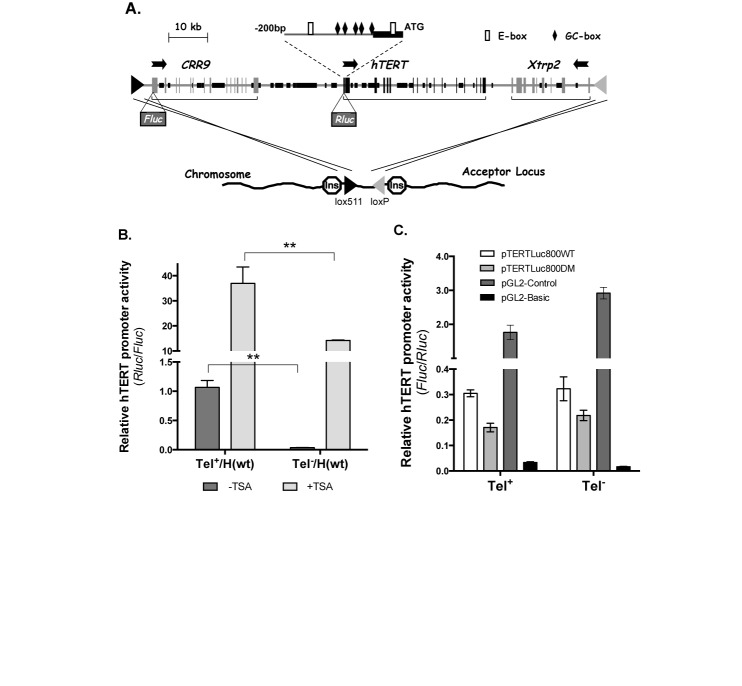
A chromatinized reporter system for hTERT transcriptional regulation. (**A**) A schematic illustration of the RMBT strategy. The top part shows the BAC reporter H(wt) containing the *hTERT*, *CRR9*, and *Xtrp2* loci. Exons are designated as vertical bars and lines. Black portions of horizontal lines represent repetitive sequences. Horizontal arrows indicate directions of transcription. *Fluc* and *Rluc* are the *Firefly* and *Renilla* luciferase expression cassettes, respectively. An expansion of the 280-bp core promoter upstream of hTERT initiation codon is shown above the diagram. The lower part shows a chromosomal acceptor site. Lox511 and loxP are represented by black and gray triangles, respectively. The acceptor locus is surrounded by a chicken β-globin insulator cHS4 (‘Ins’ in an octagon) on each side. All nucleotide positions are relative to the hTERT transcription start site (TSS). (**B**) hTERT promoter activity in a chromosomal BAC reporter H(wt) in Tel^+^ 3C167b3.1 and Tel^−^ GM847.7 lines. Luciferase activities were measured in cells from 96-well plates treated without (−TSA) or with 250nM TSA (+TSA) for 24 h. The relative activities of the hTERT promoter are shown as *Rluc*/*Fluc*. (**C**) Transient transfection of plasmid reporters. pTERTLuc800WT contains a 800-bp hTERT promoter fragment inserted into pGL2-basic reporter. pTERTLuc800DM has the same promoter fragment with mutations at two E-box sites (−165 nt and +44nt) (11). pGL2-pro and pGL-basic are control *Fluc* reporters containing an SV40 promoter and no promoter, respectively. These *Fluc* reporters, together with pRL-SV40 (a control *Rluc* reporter containing an SV40 promoter), were transfected into naïve Tel^+^ and Tel^−^ cells and luciferase activities were measured 48 h after transfection. The relative activities of transfected hTERT promoter are shown as *Fluc*/*Rluc.* **, *P* < 0.01, by Student's *t* test.

hTERT regulation is remarkably complex and has been a subject of intensive investigation for many years. The proximal region of hTERT promoter contains a number of consensus sequences, including binding sites for c-Myc, USFs, Sp1, Ets, E2Fs, AP1, HIFs and ER (10–17). Most of these transcription factors (TFs) are widely expressed and cannot fully account for specific high hTERT expression in stem cells or its activation during tumorigenesis (18). Among these sites, two E-boxes and five GC-boxes have been studied most extensively (Figure 1A). While the GC-boxes, binding sites for Sp1 family proteins, are essential for hTERT promoter function (11,13), the E-box sites play important regulatory roles (11,19,20). The canonical E-boxes (5′-CACGTG-3′), at −165 and at +44 nt, relative to the transcription start site (TSS), are binding sites for proteins of the Myc/Max/Mxd1 family and upstream stimulatory factors (USF1/2), which contain a basic helix-loop-helix (bHLH) leucine zipper structural motif (14). These sites are not only important for hTERT promoter activation by c-Myc, but also bind to Mad1 and USF1 and mediate hTERT repression (21,22). However, because these E-box binding proteins (EBPs) are widely expressed in many cell types and often bind to the same sites, it remains to be determined how they interact with one another at the hTERT promoter.

Emerging evidence has demonstrated that chromatin environment is critical for hTERT regulation in somatic cells (23,24). Inhibition of histone deacetylases (HDACs) by trichostatin A (TSA) induced hTERT transcription in these cells (24–26), indicating that the *hTERT* gene was tightly repressed. Indeed, our previous studies revealed that the *hTERT* locus was embedded in a large condensed chromatin domain in many somatic cell types (27). Given that hTERT expression was sufficient for human cell immortalization (8), stringent repression of the *hTERT* gene at its native genomic location and chromatin environment was thus a plausible explanation for the longtime observation that human cells escaped senescence and became immortalized extremely rarely (28). In other studies, transiently transfected small hTERT promoter fragments, separated from its native chromatin environment, was highly active in both telomerase-positive (Tel^+^) and -negative (Tel^−^) cells, in a stark contrast to the strongly repressed endogenous *hTERT* gene in the same host cells (23,24). Thus far, the *cis* elements that mediated hTERT regulation were all defined by transient reporter assays, which might not reflect activity of the endogenous promoter.

To study hTERT gene regulation in relevant chromatin contexts, we developed a recombination-mediated BAC targeting (RMBT) strategy to integrate bacterial artificial chromosome (BAC) constructs into pre-engineered proviral sites (29). Upon integration, the single-copy BAC construct containing the *hTERT* locus adopted a chromatin configuration similar to its native counterpart. Regulation of the hTERT promoter within the chromatinized BACs resembled that of the endogenous gene in the host cells (29).

In this report, we generated point mutations at the two canonical E-box sites within the hTERT core promoter by BAC recombineering and integrated the mutant BACs into host cells via RMBT. Thus, the mutant and wildtype hTERT promoters were studied in the same genomic and chromatin settings. By chromatin immunoprecipitation (ChIP), we found that USF1, USF2 and c-Myc/Max bound to the chromatinized hTERT promoter via these two E-boxes. Mutations at these sites reduced the hTERT promoter activity in both Tel^+^ and Tel^−^ cells, but had no effect on hTERT transcription in the presence of HDAC inhibitor TSA, suggesting that EBPs relieved the promoter repression by HDACs, likely by recruiting histone acetyl transferases (HATs) to the promoter. c-Myc overexpression activated the wildtype, but not the mutant, hTERT promoter, despite of the presence of numerous potential c-Myc binding sites downstream of the promoter in the mutant BACs (11), indicating that activation of hTERT transcription by c-Myc was mediated only by the E-box sites at core promoter. Surprisingly, knockdown (KD) of c-Myc/Max proteins also led to an induction of the hTERT promoter, independent of the two proximal E-boxes. This induction was associated with increased active histone marks, as well as increased binding of multiple TFs, at the promoter. Thus, our study revealed a new function of c-Myc protein in the regulation of hTERT gene expression: its involvement in maintaining the repressive chromatin state of the hTERT promoter. This report represented the first study of the functions of *cis* regulatory elements of the *hTERT* gene in a relevant genomic and chromatin context.

## MATERIALS AND METHODS

### BACs and plasmids

BAC 117B23-cFtRvSVP ((29) and Figure 1A), referred to as H(wt) for wildtype human BAC reporter, and plasmids phTERTLuc800WT and phTERTLuc800DM have been described previously (11,30). The E-box sites in BAC reporter H(wt) were mutated through a two-step recombineering method (31), generating H(EboxU), H(EboxD) and H(EboxDM), representing BACs containing mutations at the upstream (−165 nt), downstream (+44 nt), or both E-boxes. The mutated E-box sequences were the same as previously published (11).

### Cells, transfection and luciferase assay

Acceptor cell lines, 3C167b3.1 (Tel^+^) and GM847.7 (Tel^−^) were cultured in Minimal Essential Medium (MEM) with 10% Fetal Bovine Serum (FBS) and RMBT was performed as previously described (29). Human mammary epithelial cell line MCF10A cells were maintained in DMEM/F12 medium with 20 μg/ml insulin, 0.5 μg/ml hydrocortisone, 100 ng/ml cholera toxin and 5% horse serum. For transient transfection, hTERT reporter plasmids were transfected together with pRL-SV40 in 48-well plates using FuGene HD (Roche, Indianapolis, IN) and luciferase activities were determined 48 h post-transfection using the Dual Luciferase Assay system (Promega, Madison, WI). *Firefly* luciferase (*Fluc*) activities were normalized to *Renilla* luciferase (*Rluc*) activities from cotransfected pRLSV40. In BAC reporter assays, *Rluc* activities from the hTERT promoter were normalized to *Fluc* activities from the CRR9 promoter. In addition, the data were also normalized to cell numbers, as determined by thiazolyl blue tetrazolium bromide (MTT) assays. In all cases, the ratios of *Rluc* to *Fluc* activities correlated well with *Rluc* activities normalized to MTT counts. All reporter assays were done in triplicates and repeated at least once.

### Lentivirus preparation and shRNA KD

Lentiviral shRNA clones (Supplementary Table S1) were purchased from Sigma–Aldrich (St. Louis, MO). Control pLKO.1 plasmids, vector and scrambled shRNAs and lentiviral packaging plasmids were obtained from Addgene (Cambridge, MA). Lentiviruses were packaged in 293T cells co-transfected with a lentiviral shRNA plasmid and a cocktail of the packaging plasmids pGAG-POL, pREV and pVSVG. Lentiviral infection was performed at approximately multiplicity of infection (MOI) = 5.

### Quantitative RT-PCR and western analyses

cDNAs synthesis and quantitative polymerase chain reaction (PCR) were performed as previously described (7). Primer and probe sequences are summarized in Supplementary Table S2. For western blotting, whole cell lysates were resolved on 10% sodium dodecylsulphate-polyacrylamide gel electrophoresis and transferred to polyvinyl difluoride (PVDF) membrane, followed by detection using primary antibodies (Supplementary Table S3) and peroxidase-conjugated secondary antibodies. The western blots were visualized by SuperSignal West Femto substrate (Pierce Biotechnology, Rockford, IL) and captured with FluorChem imaging system (Protein Simple, Santa Clara, CA, USA).

### Chromatin Immunoprecipitation (ChIP)

ChIP was performed as described previously (32). Antibodies used in ChIP experiments are listed in Supplementary Table S3. Chromatin DNA fragments were subjected to quantitative PCR using primers and probes specific for the endogenous and transgenic loci (Supplementary Tables S4 and S5). Quantitative PCR reactions were performed in triplicates and the experiments were repeated at least once.

## RESULTS

### Establishment of chromatinized BAC reporters

To overcome the technical challenges rendered by chromatin dependence of hTERT regulation and to facilitate the study of hTERT promoter regulation in a chromosomal setting, we generate chromatinized hTERT reporters by integrating BAC constructs into 3C167b3.1 (Tel^+^) and GM847.7 (Tel^−^) cells using the RMBT technique (Figure 1A and (29)). Both cell lines are originated from human lung fibroblast IMR90 cells. Whereas 3C167b cells were immortalized as a result of telomerase activation, GM847 cells became immortal due to an alternative telomere lengthening (ALT) mechanism (24).

H(wt) BAC contains 160 kb of human genomic sequence encompassing three complete genomic loci, *CLPTM1L* (also called *CRR9* gene), *hTERT* and *SLC6A18* (or *Xtrp2* gene) (Figure 1A). A *Fluc* and a *Rluc* cassette were inserted at the initiation codons of *CRR9* and *hTERT* genes, respectively (29). Because *CRR9* gene is ubiquitously expressed (33), *Fluc* activity expressed from its promoter serves as an internal control. Tel^+^ and Tel^−^ are subclones of 3C167b and GM847 cells, each containing a single proviral acceptor locus. As we reported previously (29), when a single-copy H(wt) was integrated into an acceptor site, the chromatinized hTERT promoter was 50–100-folds more active in Tel^+^ cells than in Tel^−^ cells, as measured by *Rluc*/*Fluc* (Figure 1B) or by *Rluc* activities normalized to cell numbers determined by MTT assays (data not shown). The difference paralleled endogenous hTERT expression in these two cell lines (Figure 3A), but was in sharp contrast to the transiently transfected hTERT reporter plasmid, pTERTLuc800WT, which was equally active in Tel^+^ and Tel^−^ cells (Figure 1C) (23,24). pTERTLuc800WT contained a 800-bp hTERT promoter sequence upstream of the initiation codon and included most of regulatory elements based on previously reported transient transfection studies (18). Thus, RMBT-derived BAC reporters are particularly relevant for studying regulatory mechanisms involved in chromatin-dependent hTERT activation during cellular immortalization.

**Figure 2.**
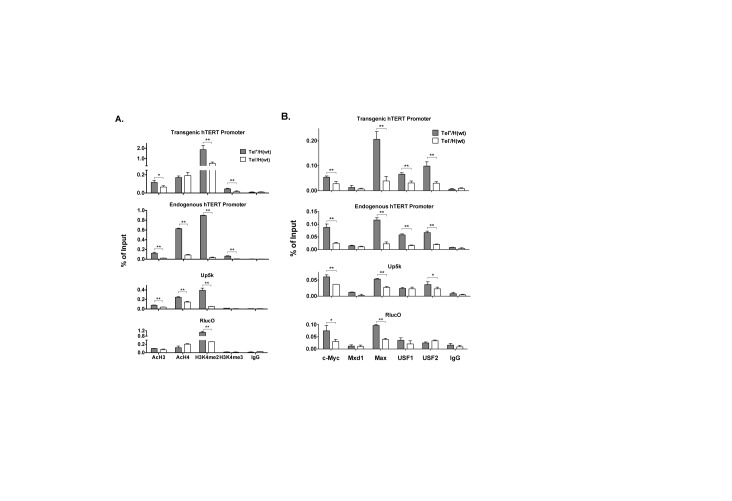
Chromatin states of integrated H(wt) BAC reporters in Tel^+^ and Tel^−^ cells. (**A**) Histone modifications at the transgenic and endogenous hTERT promoters. (**B**) Association of EBPs to the transgenic and endogenous hTERT promoters. Chromatin fragments from Tel^+^ H(wt) and Tel^−^ H(wt) cells were precipitated using antibodies against specific histone modifications (A) or EBPs (B), followed by quantitative PCR analyses. Shown are precipitated fragments as percentages of input chromatin. AcH3 and AcH3 refer to acetylated histone H3 and H4, respectively. H3K4me2 and H3K4me3 are di- and tri-methylated H3K4. Up5k and RlucO are PCR amplicons at 5-kb upstream of the hTERT promoter and 3′ end of Rluc ORF. *, *P* < 0.05; **, *P* < 0.01, by Student's *t* test.

**Figure 3.**
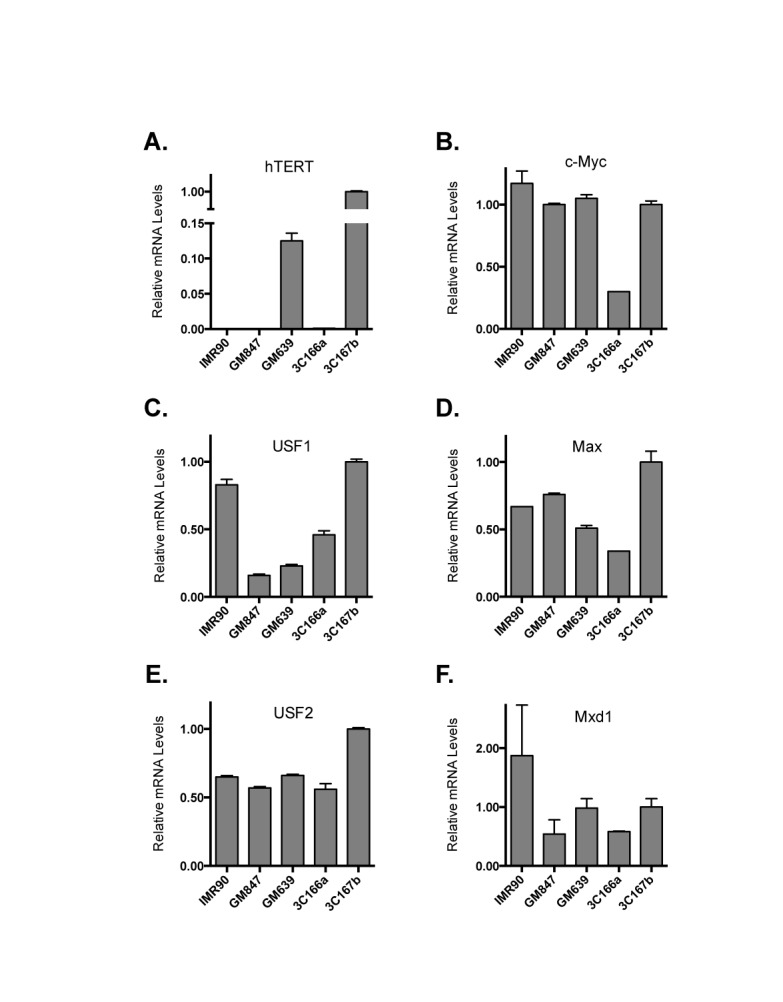
The expression of hTERT and EBPs in fibroblast lines. mRNA levels were determined by quantitative RT-PCR analyses. The y-axes show relative levels after normalized to those of 18S ribosomal RNA. IMR90, GM847 and 3C166a are telomerase-negative cells, whereas GM639 and 3C167b are telomerase-positive lines.

### Histone modifications at transgenic and endogenous hTERT promoters

Chromatin plays a critical role in the regulation of *hTERT* gene (24). As previously reported, treatment of cells with TSA, an inhibitor of classes I and II HDACs, dramatically induced the hTERT promoter activities in both Tel^+^ and Tel^−^ cells (Figure 1B), indicating that the hTERT promoter, in its native genomic contexts, was strongly repressed at least partly owing to histone deacetylation in both cell types. Thus, ChIP experiments were performed to determine the states of histone modifications for both endogenous and BAC transgenic hTERT promoter in Tel^+^ and Tel^−^ cells. PCR amplicons were designed to distinguish the transgenic and endogenous hTERT promoters. Whereas the amplicons for the endogenous and transgenic promoters shared the forward primer, the reverse primer for the transgenic promoter was positioned within the *Rluc* ORF and that of endogenous promoter was downstream of the hTERT ATG codon, thus differentiating these two promoters. In addition, PCR amplicons at genomic positions 5-kb upstream of the hTERT TSS (Up5k) and 1-kb downstream of the promoter (RlucO), positioned within the *Rluc* ORF in H(wt) BAC, were used to as negative controls. The amplicon Up5k detected both endogenous and transgenic sequences, whereas RlucO recognized only the BAC sequence.

As shown in Figure 2A, acetylation of histones H3 at both transgenic and endogenous hTERT promoters was relatively low (about or >0.1% of input), comparable to those at upstream Up5k and downstream RlucO sites, consistent with repressive status of the promoters. However, H4 acetylation at the endogenous hTERT promoter and Up5k site, but not the transgenic promoter or RlucO site, was found to be considerably higher in Tel^+^ cells than in Tel^−^ cells. This difference between the endogenous and transgenic sequences could be attributed to the presence of a translocated *hTERT* allele in Tel^+^ cells (3C167b), with the breakage point at 6-kb upstream of its promoter (30). The translocated promoter might be in a more relaxed chromatin state than the intact *hTERT* alleles since it was separated from its native chromatin environment and potential distal regulatory sequences. On the other hand, dimethylation of histone H3 lysine 4 (H3K4) at both transgenic and endogenous hTERT sequences were significantly higher in Tel^+^ cells than in Tel^−^ cells. H3K4 trimethylation was also more abundant in Tel+ cells than in Tel− cells at both transgenic and endogenous hTERT promoters, but not at Up5k and RlucO sites. Overall, these data were consistent with the finding that, while the hTERT promoter was transcriptionally active in Tel^+^ cells, the *hTERT* gene was repressed in both Tel^+^ and Tel^−^ cells.

### E-box binding proteins in immortal cell lines

Previous studies using transient reporter assays showed that multiple EBPs are involved in the regulation of *hTERT* gene (11,14). To determine whether differential expression of these proteins correlated with hTERT activation, we measured the expression of several EBPs, including USF1/2 and members of Myc superfamily proteins (Figure 3 and Supplementary Figures S1 and S2) in cell lines derived from human lung fibroblast IMR90 cells, 3C166a, 3C167b, GM639 and GM847 cells (24,34). Among these cells, 3C167b and GM639 were Tel^+^ lines that expressed hTERT mRNA, whereas 3C166a and GM847 cells are Tel^−^ ALT lines (Figure 3A). While the levels of mRNA expression of EBPs matched with their protein expression, hTERT mRNA expression did not correlate with the expression of any of EBPs among these five cell lines examined (Figure 3). For example, Tel^+^ GM639 cells expressed a lower level of USF1 and a similar level of USF2, compared to those in Tel^−^ IMR90 and 3C166a cells (Figure 3C and E). This result indicated that the abundance of EBPs by itself could not fully account for the hTERT transcription and the immortalization of Tel^+^ cells.

To determine which EBPs bound to the hTERT promoters, ChIP experiments were performed in Tel^+^ and Tel^−^ cells containing the wildtype BAC H(wt) reporter. As shown in Figure 2B, c-Myc, Max, USF1 and USF2 all bound to both transgenic and endogenous hTERT promoters more efficiently in Tel^+^ cells than in Tel^−^ cells. Conversely, Mxd1, a repressive Myc family protein, was not detected at either endogenous or transgenic hTERT promoters in these cell lines, although, as a positive control, Mxd1 signals were detected at the E-box containing NPM1 promoter in both lines (data not shown). Thus, the data showed that the hTERT promoter were occupied by multiple EBPs, contributing to its activation in Tel^+^ cells.

### E-box sites are positive elements for hTERT transcription

**Figure 4.**
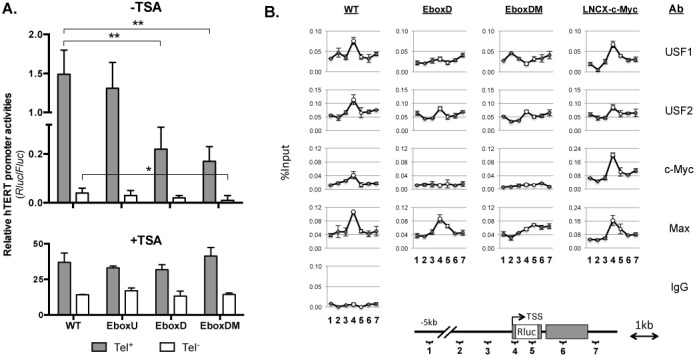
The roles of proximal E-box sites in regulating hTERT transcription. (**A**) Luciferase expression from chromatinized hTERT reporters in Tel^+^ and Tel^−^ cells. H(EboxU), H(EboxD) and H(EboxDM) are BAC reporters with point mutations at upstream (−165 nt), downstream (+44 nt) and both E-boxes at the hTERT promoter, respectively. Cells were treated without (upper chart) or with (lower chart) 250 nM TSA for 24 h prior to harvesting. (**B**) The binding of EBPs to the transgenic hTERT promoters in Tel^+^ cells. Chromatin fragments from Tel^+^ H(wt), Tel^+^ H(EboxD), Tel^+^ H(EboxDM) and LNCX-c-Myc-infected Tel^+^ H(wt) cells were precipitated with antibodies against proteins as indicated on the right, followed by quantitative PCR analysis. A diagram of genomic region spanning the hTERT promoter and positions of PCR amplicons are shown below. 1, −5kb (Up5k); 2, −2kb (Up2k); 3, −1kb (Up1k); 4, transgenic hTERT promoter; 5, Rluc ORF (RlucO); 6, +1kb (Dn1k) and 7, +2kb (Dn2k). All positions are relative to the hTERT TSS in the native genomic sequence (without the Rluc ORF). The data at the hTERT promoter are presented as large open circles; those at RlucO are indicated as small open circles; and other sites, which also occur at the endogenous hTERT loci, are shown as gray circles. The scales of y-axes of c-Myc and Max ChIP signals in LNCX-c-Myc cells are different from those of H(wt), H(EboxD) and H(EboxDM). *, *P* < 0.05; **, *P* < 0.01, by Student's *t* test.

In several previous studies using transient reporter assays, the E-box sites at the promoter were shown to function in both activation and repression of the hTERT transcription (21,22,35). To determine their regulatory roles in the genomic context and chromatin environment, point mutations were introduced at the upstream (EboxU, −165 nt,), downstream (EboxD, +44 nt), or both E-boxes (EboxDM) in H(wt) by BAC recombineering, and the resulting mutant reporters, H(EboxU), H(EboxD) and H(EboxDM), were inserted into the same acceptor lines Tel^+^ and Tel^−^ via RMBT. As shown in Figure 4A, by comparing H(wt) with H(EboxU) or H(EboxD) with H(EboxDM), EboxU mutation alone had only minor effects on hTERT transcription in both Tel^+^ and Tel^−^ cells. However, EboxD mutation in H(EboxD) and H(EboxDM) reduced hTERT promoter activities by 8–9-folds in Tel^+^ cells. This mutation also reduced hTERT promoter activity in Tel^−^ cells, although less evidently, due to the much lower hTERT expression in these cells. There seemed to be no synergistic effect of the double mutations in H(EboxDM) in either Tel^+^ or Tel^−^ cells. The data indicated that EboxD was more important for hTERT regulation while EboxU played a minor role. Upon TSA treatment, the differences between wildtype and mutant promoters disappeared (Figure 4A, lower panel), suggesting that hTERT promoters with defective E-boxes were repressed more strongly than the wildtype promoter in the same chromatin setting. These results are consistent with the notion that EboxD function to counteract hTERT promoter repression via histone acetylation or inhibition of HDACs.

Next, to determine which TFs were recruited by these E-boxes, ChIP was performed in Tel^+^ cells containing H(wt), H(EboxD) or H(EboxDM). In this experiment, in addition to the transgenic promoter region, adjacent genomic sites at Up5k, Up2k, Up1k, RlucO, Dn1k and Dn2k, were also assessed to determine the specificities of promoter recruitment of these factors. As shown in Figure 4B, the binding of USF1, USF2 and c-Myc/Max proteins to the hTERT promoter were all decreased significantly by mutating EboxD, and further diminished when both sites were mutated (EboxDM), suggesting that these proteins were recruited to the promoter via two E-boxes, particularly EboxD, at the promoter. Taken together, these data indicated that, by binding to the proximal E-box sites, the EBPs functioned to offset HDAC-mediated hTERT promoter repression.

### Effects of EBP knockdown on hTERT promoter activity

**Figure 5.**
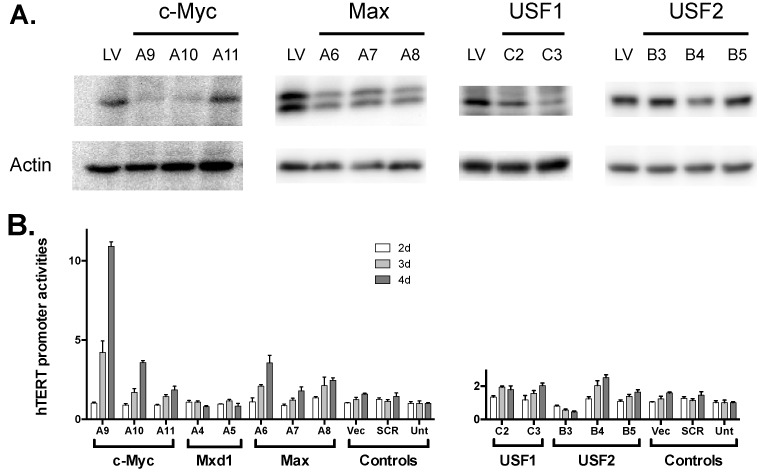
Effects of EBP knockdown on hTERT regulation. (**A**) Knockdown of EBPs by shRNAs. Tel^+^ cells were infected with lentiviral shRNAs against EBPs for 4 days. Protein expression was determined by western analyses using antibodies as indicated. Upper panels are EBPs and lower panels show actin as a loading control. (**B**) Regulation of chromatinized hTERT reporter by shRNAs. Tel^+^ H(wt) cells were infected with lentiviral shRNA and luciferase activities were measured 2, 3 and 4 days post infection. hTERT promoter activities were determined as *Rluc*/*Fluc*. Vec, lentiviral vector pLKO.1; SCR, scrambled shRNA; Unt, no infection.

To dissect the functions of individual EBPs in hTERT regulation, RNA interference was carried out using lentiviral shRNAs against each protein. In this experiment, Tel^+^ H(wt) cells were infected by individual lentiviruses and KD efficiencies were assessed by western blot analyses 4 days post infection. As shown in Figure 5A, the most efficient lentiviral shRNAs against each protein, MycA9, MycA10, MaxA6, Usf1C3 and Usf2B4, led to 50–75% inhibition of protein expression. Activity of the chromatinized transgenic hTERT promoter was determined 2–4 days following infection by lentiviral shRNAs. Surprisingly, infection by MycA9 and MycA10 led to over 10- and 4-fold increases of the hTERT promoter activity, respectively (Figure 5B). Similarly, MaxA6 also activated the hTERT promoter by 3-fold. On the other hand, shRNAs against a repressive Myc family protein, Mxd1, and two other EBPs, USF1/2, did not alter the hTERT promoter activity by more than 3-folds.

### Interplay among EBPs at the hTERT promoter

**Figure 6.**
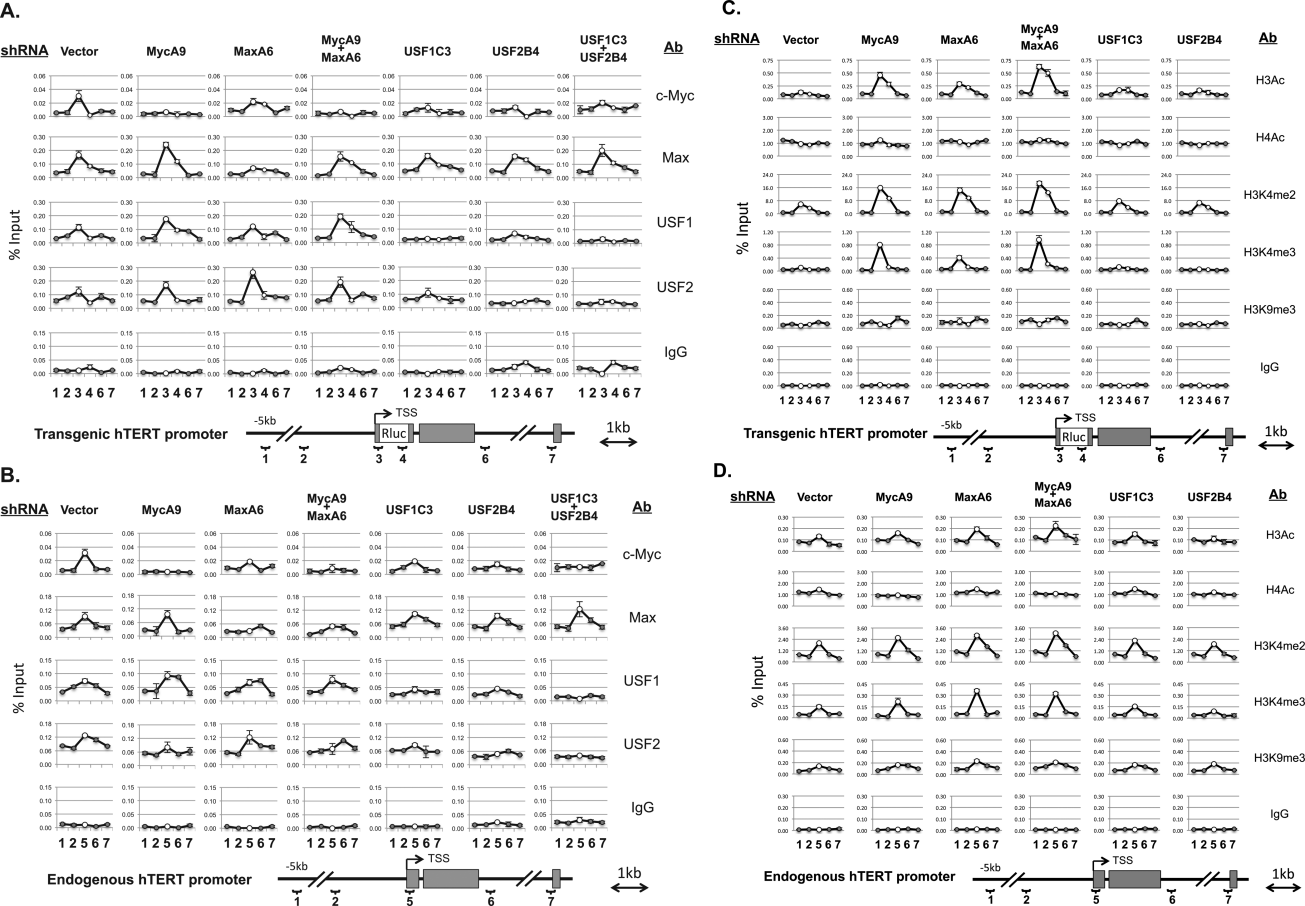
Effects of EBP knockdown on the transgenic and endogenous hTERT promoters. (**A** and **B**) Binding of EBPs to the transgenic (A) and endogenous (B) hTERT promoters. (**C** and **D**) Histone modifications at the transgenic (C) and endogenous (D) hTERT promoters. Tel^+^ H(wt) cells were infected with lentiviruses and harvested for ChIP 4 days post infection. Primer sets used in ChIP experiments are as following: 1, Up5k; 2, Up2k; 3, transgenic hTERT promoter; 4, RlucO; 5, endogenous promoter; 6, Dn2k; 7, exon 3. Amplicons 3 and 5, indicated by large open circles, were specific for the transgenic and endogenous hTERT loci, respectively. RlucO, represented by small open circles, was specific for the transgenic reporter. All other primer pairs, shown as small gray circles, recognized both transgenic and endogenous sequences.

As expected, lentiviral shRNAs against c-Myc, Max, USF1 and USF2 significantly reduced the binding of each of these proteins to the hTERT core promoters in the transgenic (Figure 6A) and endogenous (Figure 6B) loci, as determined by ChIP and quantitative PCR (qPCR) analyses. The relatively weak signal of c-Myc binding was likely due to the antibody used in this experiment, although this antibody was the best among the four c-Myc antibodies we tested (data not shown).

To understand the interplay among these EBPs at the hTERT promoters, promoter recruitment of all proteins was measured upon the KD of each EBP. Not surprisingly, Max KD attenuated c-Myc binding to the transgenic hTERT promoter (Figure 6A) as c-Myc/Max was predicted to bind to E-boxes as a heterodimer. However, c-Myc KD unexpectedly increased Max binding to the transgenic hTERT promoter. A likely explanation was that Max bound to the E-boxes sites as a homodimer or by forming heterodimers with other Myc family proteins when the level of c-Myc protein was reduced (36). In addition, knocking down c-Myc alone or both c-Myc and Max increased the association of USF1 and USF2 to the transgenic hTERT promoter (Figure 6A), and to a lesser extent, to the endogenous promoter (Figure 6B). Therefore, c-Myc KD resulted in increased recruitment of multiple EBPs to the hTERT promoter.

On the other hand, KD of USF1, USF2 or both proteins led to a decrease in the binding of c-Myc, but not Max, to transgenic and endogenous hTERT promoters (Figure 6A and B), although c-Myc protein expression was not affected significantly in these cells (Supplementary Figure S3). Further, knocking down USF1 or USF2 also reduced one another's association to the hTERT promoters, consistent with their ability to bind DNA as a heterodimer (37). By and large, the interplay of EBPs at the hTERT promoter was rather complex. Their abilities to bind DNA either as homodimers or as heterodimers might have contributed to this complexity. In addition, it was also possible that recruitment of EBPs to the hTERT promoter modulated its chromatin accessibility (38) and consequently impacted one another indirectly.

### Effects of EBP knockdown on histone marks at the hTERT promoter

To determine chromatin changes associated with reduced recruitment of EBPs to the hTERT promoter, covalent modifications of histones H3 and H4 were examined by ChIP/qPCR analysis. As shown in Figure 6C and D, individual lentival shRNAs against c-Myc and Max increased H3 acetylation at both the transgenic and endogenous hTERT promoters; the increase was more profound at the transgenic promoter (Figure 6C) than the endogenous promoter (Figure 6D). Likewise, the active histone marks of transcription, di- and tri-methylation of H3K4, also increased upon KD of c-Myc and Max. H4 acetylation maintained relatively constant throughout the 10-kb genomic region surrounding the hTERT promoter, whereas H3K9 trimethylation, a repressive histone mark, remained low in the same region. KD of c-Myc/Max did not have significant effects on these two epigenetic marks. Thus, c-Myc/Max KD resulted in increases of several active histone marks at the hTERT promoter.

### c-Myc knockdown increased recruitment of multiple TFs

**Figure 7.**
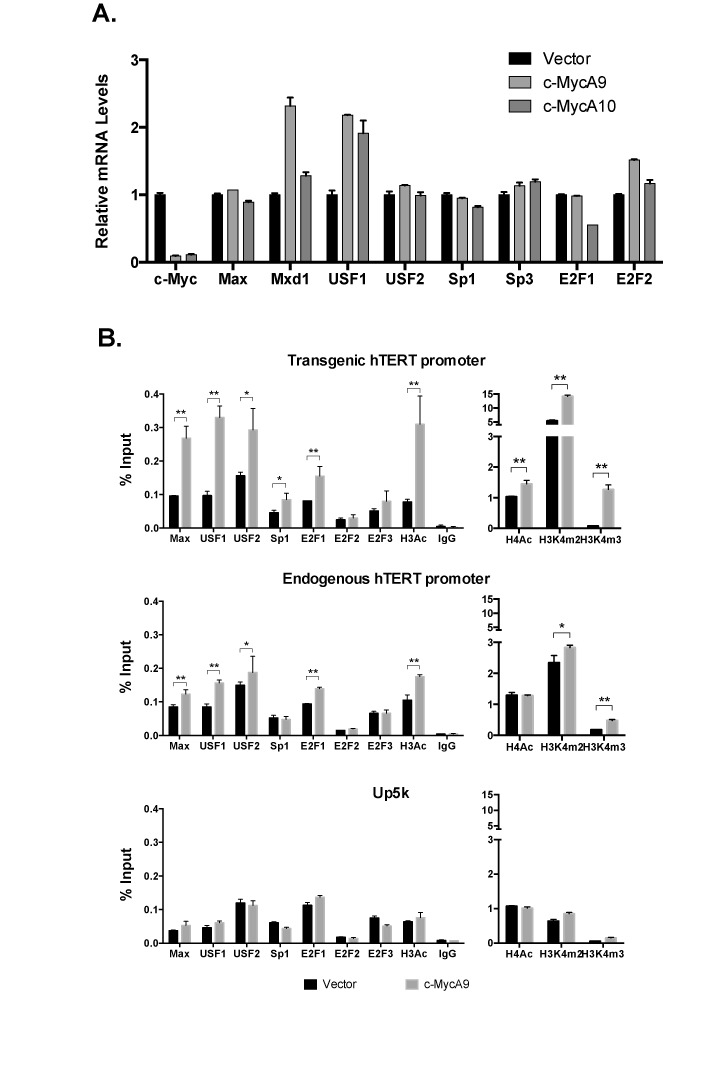
Effects of c-Myc KD on TF recruitment to the hTERT promoters. Tel^+^ H(wt) cells were infected with lentiviral shRNAs against c-Myc, c-MycA9 and c-MycA10, for 4 days. (**A**) The expression of TFs. mRNA levels were measured by quantitative RT-PCR and normalized to 18S rRNA. (**B**) Binding of TFs to the hTERT promoters. ChIP experiments were performed as described in Figure 6. *, *P* < 0.05; **, *P* < 0.01, by Student's *t* test.

Because c-Myc/Max was found to function as activators of the hTERT promoter (11,39), it was a surprise to discover that knocking down c-Myc/Max in fact activated the hTERT promoter. One explanation is that c-Myc/Max KD indirectly activated the hTERT promoter, by activating or inhibiting other regulatory factors of the hTERT promoter. Yet, qRT-PCR and western analyses showed that c-Myc KD did not lead to induction of mRNA or protein levels of USF1, USF2, Sp1, E2F1 and E2F2 (Figure 7A and Supplementary Figure S4), all of which have previously been reported to activate hTERT transcription (11,14,40,41). On the other hand, the mRNA levels of potential repressive TFs, Mxd1 and Sp3, were not decreased upon c-Myc KD (Figure 7A). However, an increased binding to the transgenic hTERT promoter and, to a lesser extent, the endogenous promoter was observed for nearly all of the active TFs tested (Figure 7B). As a control, TF recruitment and active histone marks were unaffected at Up5k, indicating that c-Myc KD induced chromatin changes was localized to the hTERT promoter. Taken together, accumulation of active histone marks, along with increased promoter binding by multiple TFs, as a result of c-Myc KD, suggested a model that reducing the c-Myc level led to an opening of the chromatin configuration at the hTERT promoter and an increase of its accessibility to TF binding.

### Proximal E-boxes were not required in hTERT activation upon c-Myc knockdown

**Figure 8.**
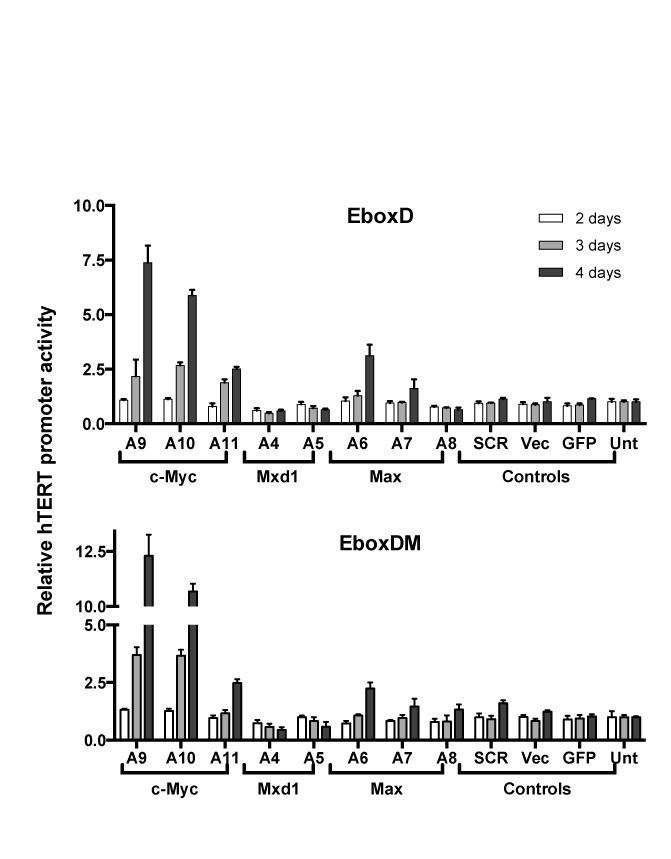
Effects on mutant hTERT promoters by EBP knockdown. Tel^+^ H(EboxD) and Tel^+^ H(EboxDM) cells were infected with lentiviral shRNAs. Luciferase activities were measured 2, 3 and 4 days after infection. hTERT promoter activities were determined as *Rluc*/*Fluc*.

Previous studies, using transiently transfected promoter fragments, have shown that the two proximal E-boxes were critical in c-Myc mediated hTERT activation (42). To determine whether these sites were directly involved in the modulation of chromatinized hTERT promoter functions, Tel^+^ cells containing H(EboxD) or H(EboxDM) were infected with lentiviral shRNAs against c-Myc as well as other Myc family proteins Max and Mxd1. As shown in Figure 8, the mutant hTERT promoters in both H(EboxD) and H(EboxDM) were induced upon knocking down c-Myc and Max, but not Mxd1, similar to the wildtype promoter in H(wt) (Figure 5B), indicating that these E-boxes were dispensable for the hTERT activation by c-Myc/Max KD.

**Figure 9.**
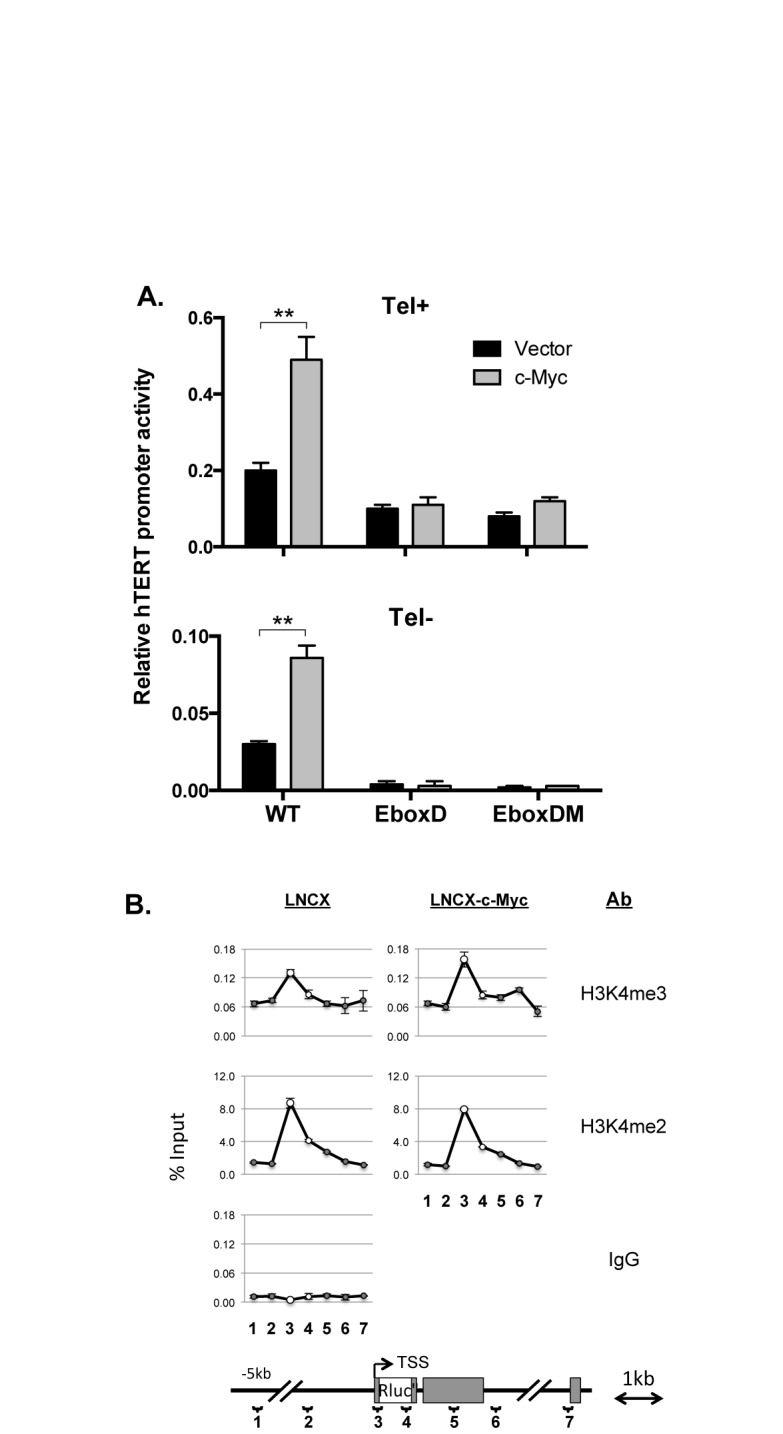
Requirement of proximal E-box sites for hTERT activation by c-Myc overexpression. (**A**) hTERT promoter activation by c-Myc overexpression. Tel^+^ and Tel^−^ cells containing integrated BAC reporters H(wt), H(EboxD) or H(EboxDM) were infected with LNCX-c-Myc or LNCX vector retroviruses. Luciferase activities were measured in stable neomycin-resistant pools and hTERT promoter activities were determined as *Rluc*/*Fluc* ratios. (**B**) H3K4 Methylation at the transgenic hTERT promoter in c-Myc overexpressing cells. Primer sets are: 1, Up5k; 2, Up2k; 3, transgenic hTERT promoter; 4, RlucO; 5, Dn1k; 6, Dn2k; 7, exon 3. *, *P* < 0.05; **, *P* < 0.01, by Student's *t* test.

To determine whether these E-boxes were required for hTERT promoter activation by c-Myc overexpression, pLNCX-c-Myc, a retrovirus expressing c-Myc (27), was transduced into Tel^+^ and Tel^−^ cells. c-Myc expression was 3–5-fold higher in c-Myc virus infected cells than in control cells as determined by western analysis (Supplementary Figure S4). c-Myc overexpression in Tel^+^ H(wt) cells led to a significantly increased recruitment of c-Myc/Max, but not USF1 or USF2, to the hTERT promoter (Figure 4B, right column). In both Tel^+^ and Tel^−^ cells, c-Myc activated the hTERT promoter in H(wt) by 2–3-folds, but failed to activate the hTERT promoters in H(EboxD) and H(EboxDM) reporters (Figure 9A). This activation by c-Myc overexpression was accompanied by an increase in H3K4 trimethylation, but not its dimethylation, at the hTERT promoter, in Tel^+^/H(wt) cells (Figure 9B). Together, these data indicated that c-Myc mediated hTERT activation required the proximal E-boxes, particularly the downstream E-box, in a chromosomal environment similar to the native states of *hTERT* gene.

### Regulation of endogenous hTERT expression

**Figure 10.**
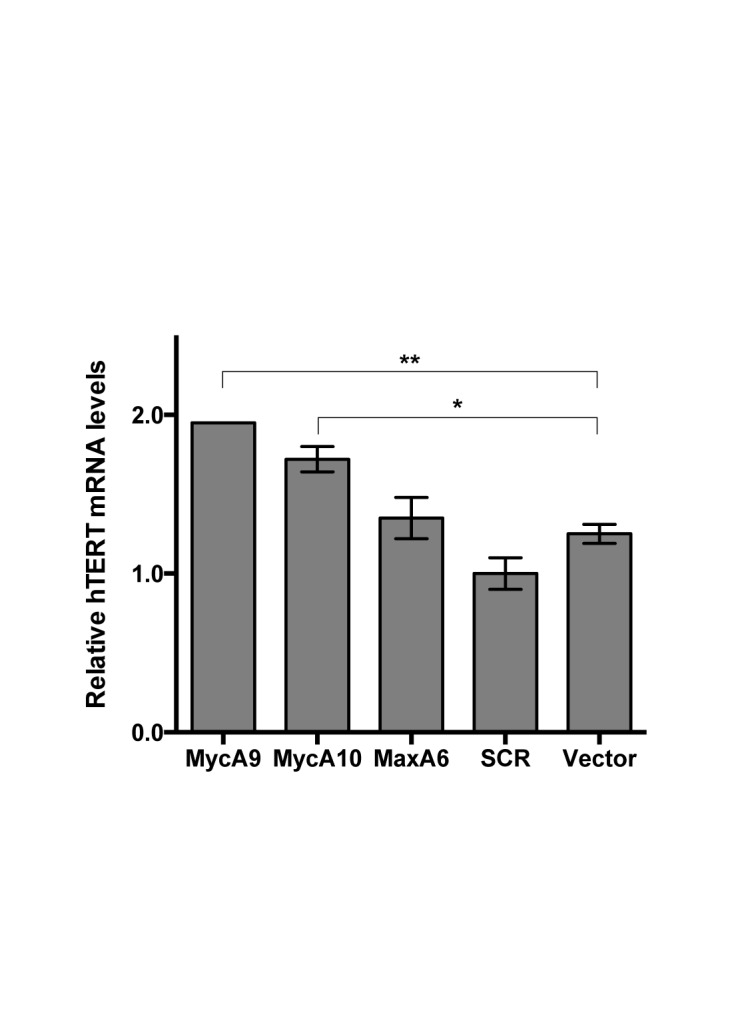
Effect of c-Myc/Max knockdown on hTERT expression in human epithelial cell line MCF10A. MCF10A cells were infected with lentiviral shRNAs and selected with 1 μg/ml puromycin for 4 days. hTERT mRNA levels were determined by quantitative RT-PCR and normalized to 18S rRNA. *, p < 0.05; **, p < 0.01, by Student's* t* test.

While hTERT activation by c-Myc overexpression has been previously reported, it was a surprise to discover that c-Myc/Max KD also activated hTERT transcription in a chromosomal context in fibroblasts. To substantiate this finding, we used an immortal, but untransformed, human breast epithelial cell line MCF10A. Lentiviral shRNAs against c-Myc and Max were transduced into MCF10A cells. As shown in Figure 10, lentiviruses MycA9 and A10, containing the two most potent shRNAs against c-Myc and Max, induced endogenous hTERT mRNA expression by nearly 2-fold four days post infection. Western analysis confirmed that the lentiviral shRNAs inhibited the expression of their respective target proteins (Supplementary Figure S5). In addition, the lentiviruses were also transduced into telomerase-positive transformed fibroblasts GM639 and breast cancer cells T-47D, resulting in the induction of endogenousl hTERT mRNA in these cells. Again, the induction correlated with KD of c-Myc or Max mRNAs (Supplementary Figure S6). Thus, depletion of c-Myc/Max TF complex also induced endogenous hTERT transcription in immortal fibroblasts and epithelial cells, indicating that RMBT created hTERT reporter system provided an advantageous platform for studying the complex hTERT transcriptional regulation.

### c-Myc expression modulates hTERT promoter induction by shRNAs

**Figure 11.**
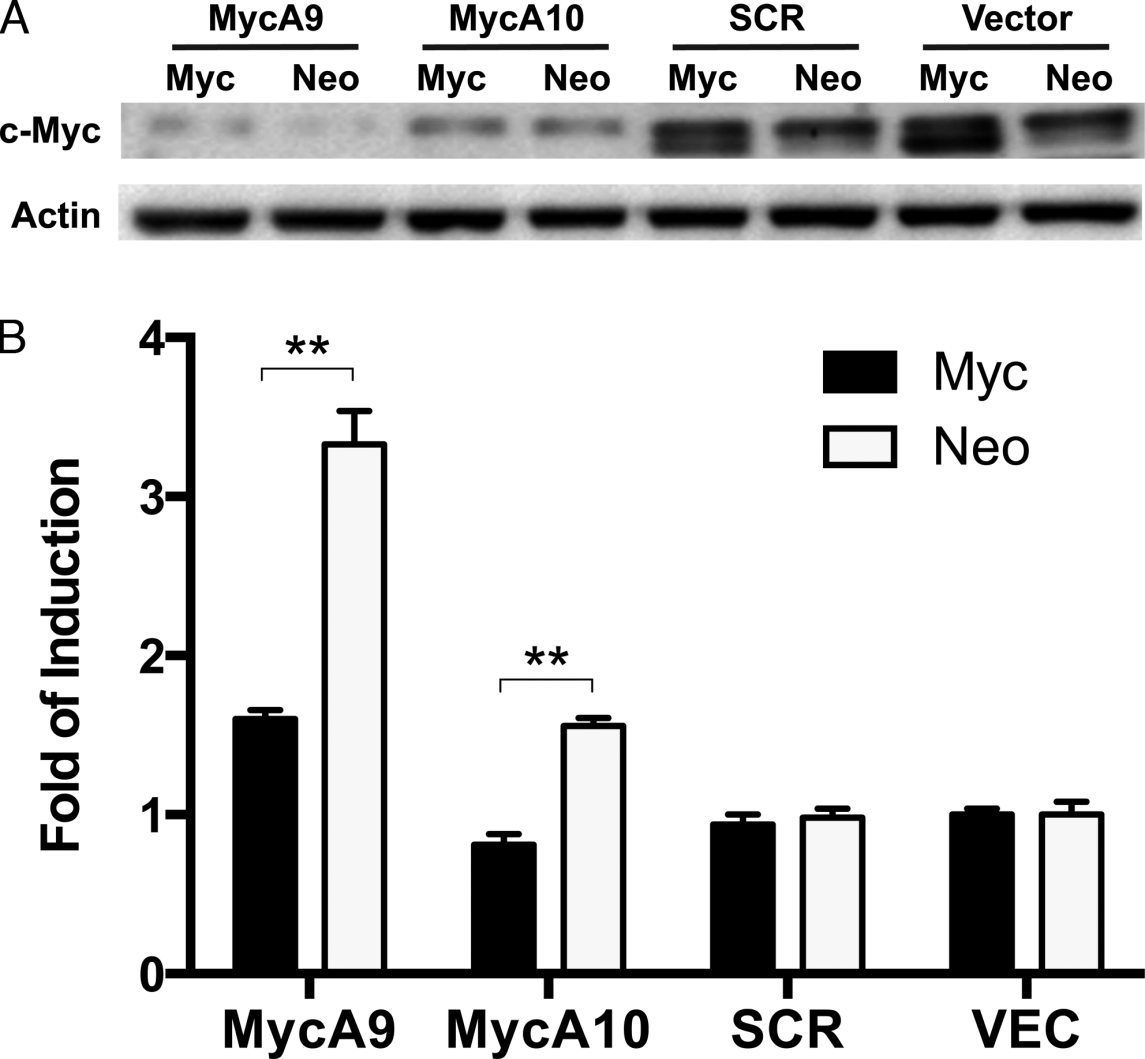
Effect of c-Myc overexpression on hTERT promoter induction. (**A**) c-Myc KD in Tel^+^/H(wt) cells containing LNCX-c-Myc (Myc) or LNCX vector (Neo) retroviruses. The cells were transduced with lentiviral shRNAs and harvested for western analysis 4 days later. (**B**) Induction of transgenic hTERT promoter. Luciferase activities were measured 4 days post infection and hTERT promoter activities were determined as *Rluc*/*Fluc*. **, *P* < 0.01, by Student's* t* test.

To further demonstrate that low c-Myc expression resulted in the activation of hTERT promoter, Tel^+^/H(wt) cells containing LNCX-c-Myc or LNCX vector were infected with lentiviral shRNAs. As shown in Figure 11, MycA9 and MycA10 inhibited the expression of both endogenous and retroviral c-Myc proteins. However, the residual c-Myc expression was higher in LNCX-c-Myc transduced cells than in vector cells. As a result, MycA9 and MycA10 in c-Myc-expressing cells led to a weaker or no induction of the hTERT promoter, respectively. The data was consistent with the conclusion that inhibition of c-Myc expression led to the hTERT activation.

## DISCUSSION

Chromatin-dependent repression is at the heart of hTERT regulation in somatic cells and transient reporter assays are not sufficient for deciphering its mechanisms (24). Hence, we developed the RMBT method to integrate single-copy BAC reporters into pre-engineered chromosomal acceptor sites (29), allowing large intact BAC reporters to acquire relevant chromatin configurations. Site-specific integration also minimizes the impact of chromosomal positional effects on the integrated reporters. Indeed, the chromatinized hTERT promoter in BAC reporter H(wt) recapitulated its endogenous counterparts in both Tel^+^ and Tel^−^ cells (29). This method, together with mutagenesis by BAC recombineering (31), constitutes an effective system for dissecting *cis* elements involved in chromatin-dependent hTERT repression.

In this report, we focused on the two canonical E-boxes at the hTERT core promoter. Our results demonstrated that these sites were required for recruiting EBPs to the promoter and for activating hTERT transcription by overexpressed c-Myc. When these sites were mutated, the hTERT promoter became even more strongly repressed by 8–9-folds and this strengthened repression was reversed by HDAC inhibition (Figure 4A), suggesting that EBPs acted to de-repress the hTERT promoter. In contrast, the same mutations reduced hTERT promoter activity by only 30% in transiently transfected plasmid reporters containing an 800-bp hTERT promoter fragment (Figure 1C). These data indicated that the anti-repression effect of the E-boxes required a pertinent chromatin environment. As we reported previously, the *hTERT* locus was embedded in a nuclease-resistant chromatin domain in both Tel^+^ and Tel^−^ cells (27). In this native context, the E-boxes may function to de-repress the hTERT promoter, thereby allowing its transcription in Tel^+^ cells.

Because proto-oncogene c-Myc is frequently overexpressed in cancers, hTERT activation by c-Myc via the proximal E-boxes at the hTERT promoter is likely important for tumorigenesis. It has been reported that c-Myc activates transcription through several mechanisms (43). c-Myc may recruit chromatin modifying complexes, including those containing Transformation/Transcription Domain-Associated Protein (TRRAP) and associated histone acetylases (HATs). In addition, it can also recruit P-TEFb, a kinase that phosphorylates the carboxy-terminal domain (CTD) of PolII, and regulates promoter clearance or elongation of a stalled pre-initiation complex. Although we have not tested whether the E-boxes affected promoter clearance in our experimental settings, the data revealed a major role for c-Myc to counteract against HDAC-dependent hTERT promoter repression, suggesting that recruitment of HAT-containing complexes by c-Myc was important for hTERT de-repression.

Telomere maintenance as a result of hTERT expression is a hallmark of cancer (44) and the key to activate *hTERT* gene is likely to relieve its chromatin-dependent repression, or de-repression (18). Besides the de-repression owing to c-Myc overexpression, the hTERT promoter also suffers genetic mutations in cancer cells. In melanoma, familial and recurrent somatic mutations at the hTERT promoter created *de novo* binding sites for Ets/TCF TFs, a downstream target of Braf mutations in the patients (45,46). Thus, the mutated hTERT promoters were activated by these TFs and associated HAT-containing CBP/p300 complexes (47).

A surprising finding of this study is the activation of the hTERT promoter upon c-Myc/Max KD. This activation, however, was independent of the proximal E-boxes, because the hTERT promoter in H(EboxD) and H(EboxDM), in which the E-boxes were mutated, were similarly activated. c-Myc has been previously shown to repress several negative regulators of cell proliferation, including c/EBPα, p15Ink4b, p21Cip1 and p27Kip1, by interacting with zinc finger TFs Sp1 and Miz1 that bound to initiator elements of TATA-less promoters of the target genes (48). Thus, we considered the possibility that c-Myc might possess dual functions in regulating the hTERT promoter: activation by binding to the E-boxes and repression via the initiator element of the hTERT promoter. While we could not completely rule out this possibility, our data showed that over-expressed c-Myc did not repress the mutant hTERT promoters in H(EboxD) and H(EboxDM) (Figure 9A). Although c-Myc/Max did not bind the mutant hTERT promoters in these BAC reporters, c-Myc/Max KD still induced these promoters (Figure 4B), suggesting that c-Myc/Max might repress the hTERT promoter indirectly. Indeed, the activation of hTERT promoter by c-Myc KD was accompanied by increased promoter accessibility, as indicated by elevated binding of multiple TFs, including USF1/2, Sp1 and E2F family proteins, to the promoter. Therefore, our data were consistent with the model that c-Myc/Max KD opened chromatin at the hTERT promoter, rendering it more accessible to multiple TFs.

Most studies have shown that Myc family proteins regulate gene expression via direct promoter binding. However, this notion was challenged by the finding that loss of myc function resulted in changes in global chromatin structure, accompanied by histone hypoacetylation and altered methylation (38). While Knoepfler et. al. showed that loss of myc led to nuclear condensation (38), our current study demonstrated that c-Myc/Max KD resulted in increased hTERT promoter accessibility. Thus, both studies indicated that Myc function was important for maintaining chromatin structural integrity.

Whereas the activity of transgenic hTERT promoter paralleled that of the endogenous promoter, chromatin changes in response to c-Myc/Max KD were greater at the transgenic promoter. This was not a complete surprise because the endogenous genes have been subjected to epigenetic modifications during developmental processes as well as *in vitro* cell proliferation, whereas the transgenic loci were established by *de novo* assembly of chromatin on the integrated BAC DNAs. For example, H3K9 trimethylation was somewhat higher at the endogenous promoter than the transgenic reporter (Figure 6C and D) and this might have contributed to the stronger repression of endogenous *hTERT* loci. In addition, most of the epigenetic differences between transgenic and endogenous loci are difficult to detect, due to their identical DNA sequences. Nevertheless, while potential differences between epigenetic states of endogenous and transgenic loci might impact on their regulation, our study demonstrated that the chromatinized hTERT reporters are much-improved models for studying chromatin-dependent hTERT regulation than transient reporter assays.

In summary, we report here the first mutational analysis of DNA elements in a chromatin context using the RMBT technique. These experiments demonstrated, for the first time, that c-Myc/Max had dual functions in hTERT regulation, that is, its involvement not only in activating hTERT transcription but also in maintaining the repressive states of hTERT promoter in somatic cells.

## SUPPLEMENTARY DATA

Supplementary Data are available at NAR Online.

SUPPLEMENTARY DATA
