# Supplementary material for: Dual roles of c-Myc in the regulation of hTERT gene
Source: Nucleic Acids Res. 2014 Aug 28;42(16):10385–98. doi: 10.1093/nar/gku721 (PMC4176324; doi:10.1093/nar/gku721)
Supplement: SUPPLEMENTARY DATA [file supp_42_16_10385__index.html]

Dual roles of c-Myc in the regulation of hTERT gene — Dual roles of c-Myc in the regulation of hTERT gene — SUPPLEMENTARY DATA 

# Dual roles of c-Myc in the regulation of *hTERT* gene

## SUPPLEMENTARY DATA

**Files in this Data Supplement:**

- SUPPLEMENTARY DATA
- SUPPLEMENTARY DATA
- SUPPLEMENTARY DATA
