# Supplementary figures and images for: Dual roles of c-Myc in the regulation of hTERT gene
Source: Nucleic Acids Res. 2014 Aug 28;42(16):10385–98. doi: 10.1093/nar/gku721 (PMC4176324; doi:10.1093/nar/gku721)

Figure S1

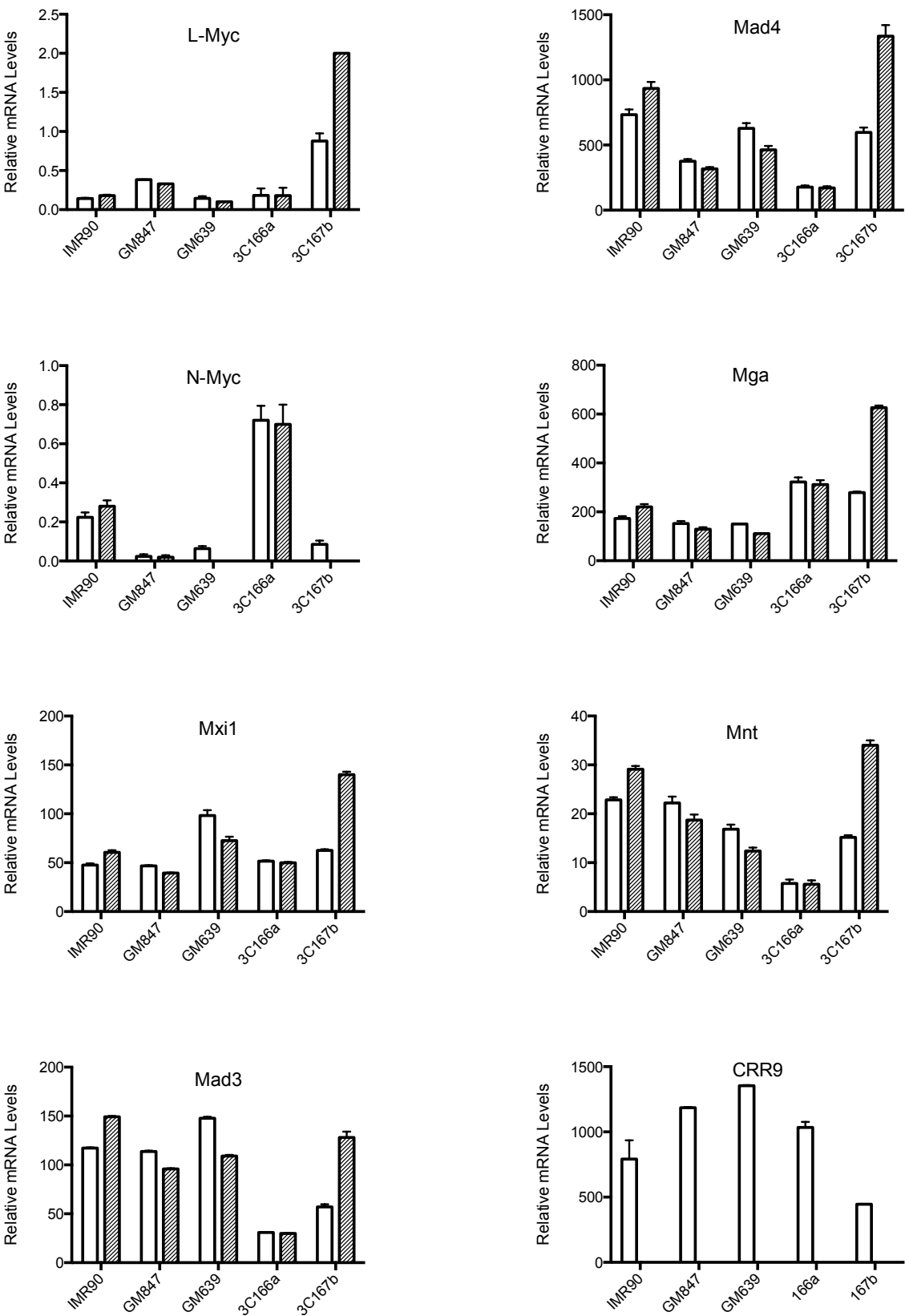

*Figure S2*

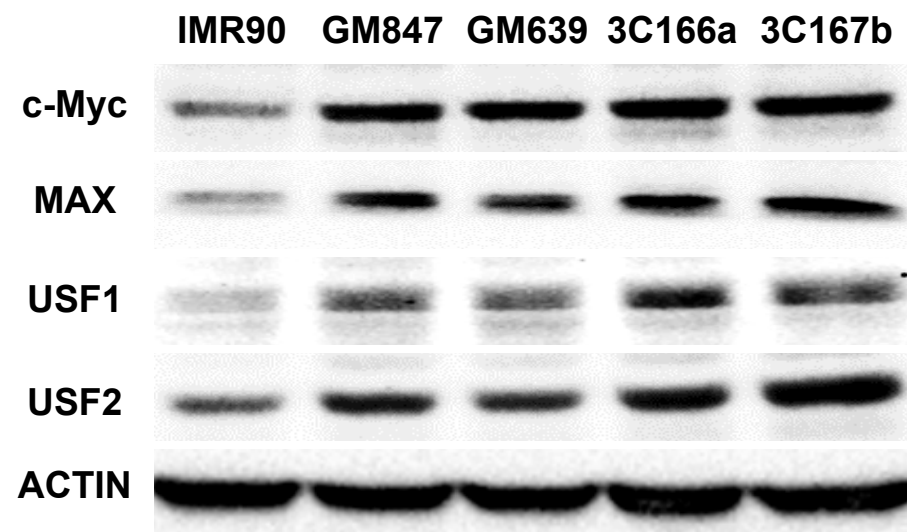

*Figure S3*

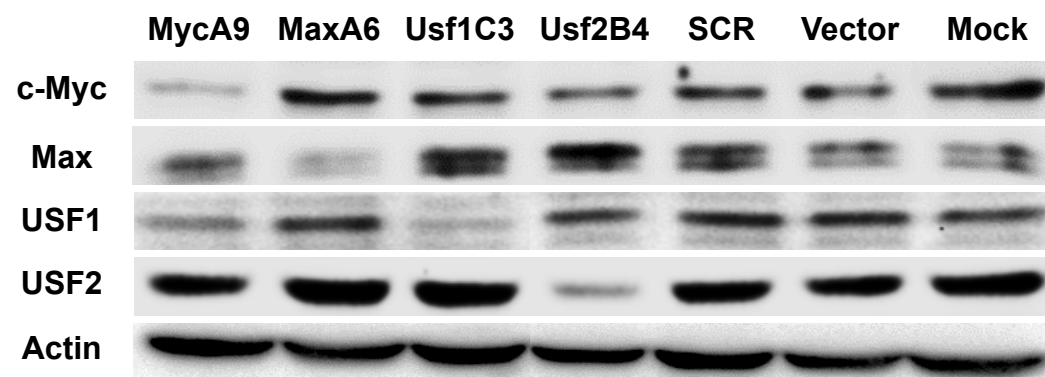

Figure S4

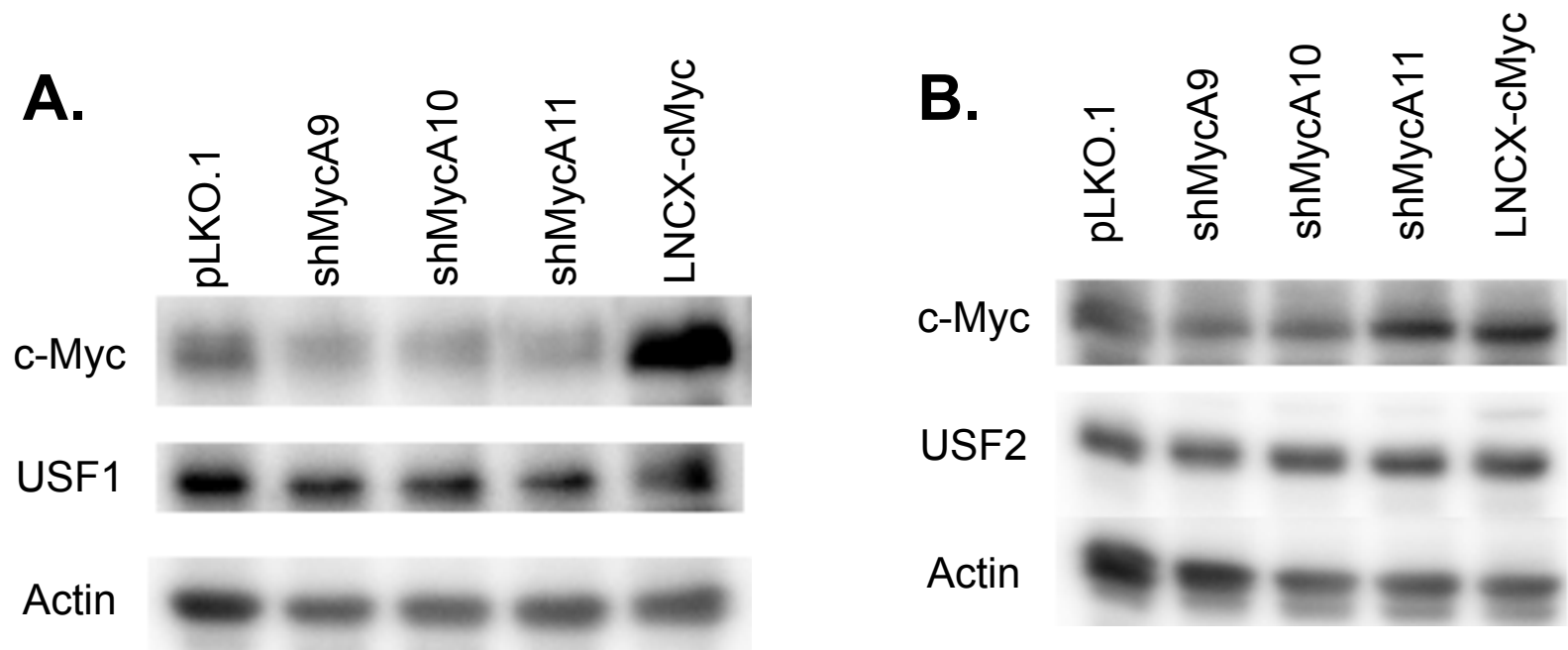

*Figure S5*

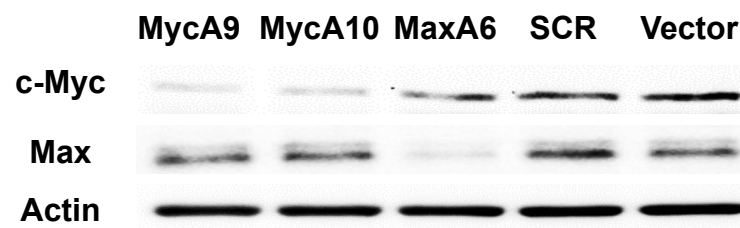

Figure S6

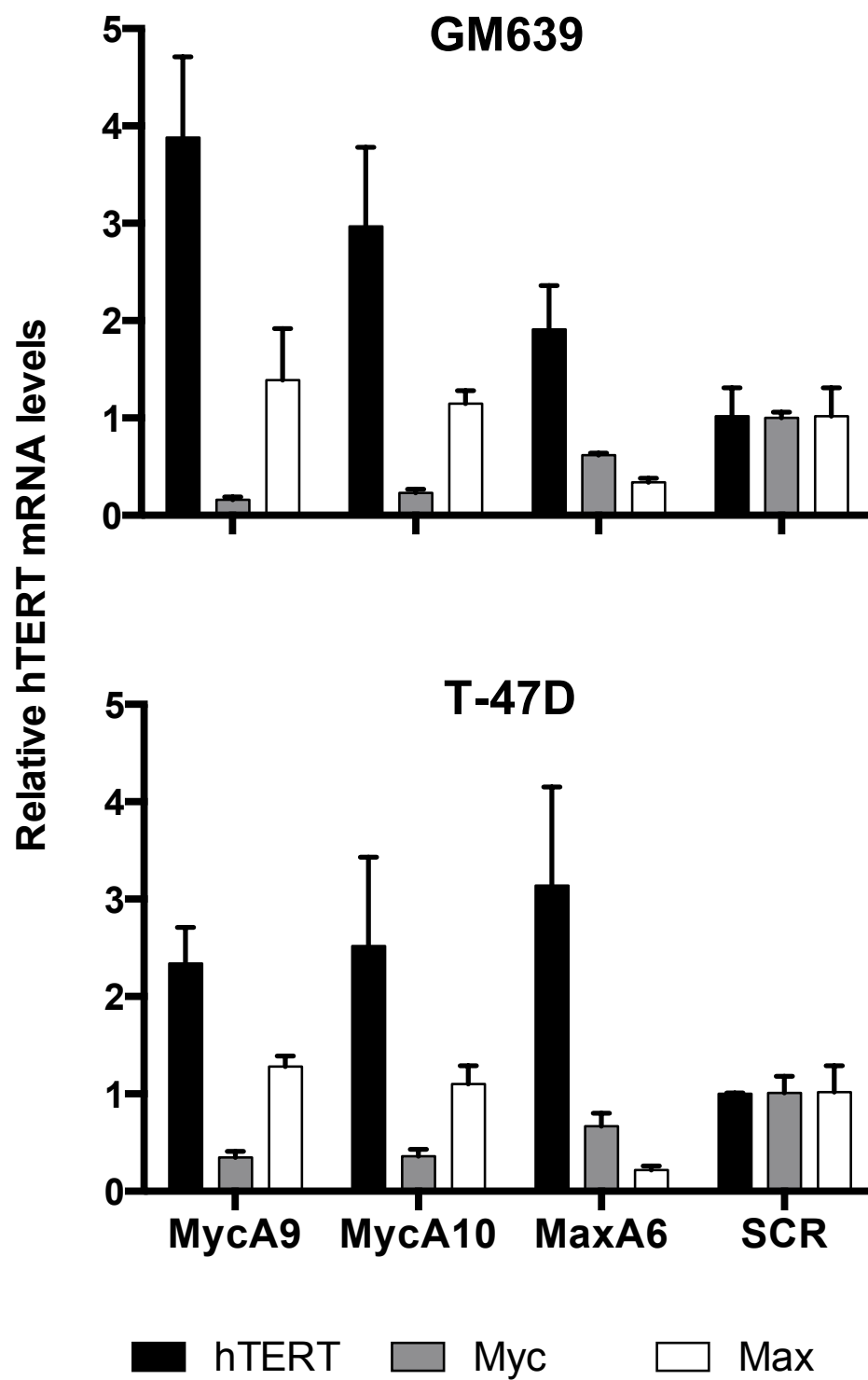

Supplement: SUPPLEMENTARY DATA [file supp_gku721_nar-00016-x-2014-File003.pdf]
