# Supplementary material for: Dual roles of c-Myc in the regulation of hTERT gene
Source: Nucleic Acids Res. 2014 Aug 28;42(16):10385–98. doi: 10.1093/nar/gku721 (PMC4176324; doi:10.1093/nar/gku721)
Supplement: SUPPLEMENTARY DATA [file supp_gku721_nar-00016-x-2014-File005.docx]

Figure S1. Expression of Myc family TFs in fibroblast cells. Quantitative RT-PCR analyses were performed as described in Figure 3. Grey and white bars represent relative mRNA levels normalized to 18S rRNA and CRR9, respectively.

Figure S2. Expression of E-box binding proteins in fibroblast lines. Total proteins (10µg) were examined by Western blot analyses.

Figure S3. Effects of individual EBP knockdown on the expression of other EBPs. Tel+ cells were infected as described in Figure 5 and harvested 4 days post infection.

Figure S4. Effects on USF1 and USF2 protein expression by c-Myc KD. Tel+ cells were infected as described in Figure 5 and harvested 4 days post infection.

Figure S5. Expression of c-Myc and Max proteins in IMR90 cells.

Figure S6. Induction of endogenous hTERT mRNA upon KD of c-Myc and Max. GM639 fibroblasts and T-47D breast cancer cells were transduced with lentiviruses and total RNAs were harvest 4 days post infection. The levels of hTERT, c-Myc, and Max mRNA were determined by qRT-PCR and normalized to 18S rRNA.
